# Demographic profiles and extent of critical care resources utilisation in patients with severe traumatic brain injury: a Tan Tock Seng Hospital National Neuroscience Institute study

**DOI:** 10.1186/cc13667

**Published:** 2014-03-17

**Authors:** J Yang, JZ Wee, CT Chong

**Affiliations:** 1 Tan Tock Seng Hospital, Singapore; 2 Ministry of Health, Singapore

## Introduction

Trauma is the fifth principal cause of death in Singapore, with traumatic brain injury (TBI) being the leading specific subordinate cause [1].

## Methods

This 8-year retrospective review of patients with severe TBI admitted to the neuroICU (NICU) of the National Neuroscience Institute, Tan Tock Seng Hospital between 2004 and 2011 reports the demographic profiles of severe TBI in the local context, with implications in the management of severe TBI, particularly the utilisation of critical care resources.

## Results

A total of 780 patients were admitted with TBI during the study period, of which 365 patients (46.8%) sustained severe TBI. The majority (75.3%) of the severe TBI patients were male. There was a bimodal preponderance of severe TBI cases in young adults (age 21 to 40) and older people (age >61). Motor vehicle accidents (48.8%) and falls from <2 m (35.1%) were the main mechanisms of injury. Invasive monitoring was frequently employed in these patients with severe TBI: arterial blood pressure monitoring in 298 patients (81.6%), central venous pressure monitoring in 219 patients (60.0%), and intracranial pressure monitoring in 173 patients (47.4%). The incidence of use of tiered therapy such as sedation, mild hyperventilation, osmotherapy with mannitol, cerebrospinal fluid drainage, barbiturate coma and decompressive craniectomy to control ICP converged with international practices.

## Conclusion

Young adults and older people involved mainly in motor vehicle accidents and falls respectively were among the high-risk groups for severe TBI. Management of these patients goes beyond the ICU and involves, but is not limited to, social support, emotional motivation and community reintegration of these patients [2]. TBI among the high-risk groups is largely preventable. Public awareness and prevention programmes will go some way in reducing the incidence of TBI amongst the high-risk groups.
